# Association of Toll-Like Receptor 2 (TLR2) Expression in Placenta and Intrauterine Exposure to Hepatitis B Virus

**DOI:** 10.1155/2022/4838376

**Published:** 2022-07-13

**Authors:** Dian Utami, Maisuri T. Chalid, Rina Masadah, Rizalinda Sjahril, Andi Dwi Bahagia Febriani

**Affiliations:** ^1^Department of Obstetrics and Gynecology, Faculty of Medicine, Hasanuddin University, Makassar, Indonesia; ^2^Department of Pathology Anatomy, Faculty of Medicine, Hasanuddin University, Makassar, Indonesia; ^3^Department of Microbiology, Faculty of Medicine, Hasanuddin University, Makassar, Indonesia; ^4^Department of Pediatrics, Faculty of Medicine, Hasanuddin University, Makassar, Indonesia

## Abstract

**Introduction:**

The placenta is a specialized organ that only performs during pregnancy and serves as an immunological barrier in preventing pathogens to reach the fetus. It has been known that toll-like receptors (TLRs) on the placenta respond to antigens, such as zymosan, lipopolysaccharide, and other viral infections. This study analyzes the distribution of TLR2 protein and hepatitis B DNA virus (HBV DNA) virus identification to gain an understanding of hepatitis B viral transmission from the mother to child.

**Methods:**

We performed enzyme-linked immunoadsorbent assay of HBeAg, HBsAg titer, Anti-HBs, and Anti-HBc to 59 HBsAg-positive pregnant women and identified HBV DNA using nested PCR in their cord blood during delivery for evidence of exposure to HBV DNA. The expression of TLR2 protein in the placenta was performed using immunohistochemistry analysis.

**Results:**

Intrauterine exposure to the hepatitis B virus occurred in 69.5% of all pregnant women who were HbsAg positive. TLR2 expression was predominantly identified in syncytiotrophoblast and cytotrophoblast cells with the highest score in mothers aged 20–35 years (75%), multigravida (58.3%), and mothers with term pregnancies (70.8%). Statistical analysis results showed that placental TLR2 expression did not indicate any association with hepatitis B virus DNA identified in cord blood with a *p* value of 0.730 and an OR of 0.650 (95% CI 0.173–2.440).

**Conclusion:**

TLR2 expression is not associated with intrauterine exposure of hepatitis B virus.

## 1. Introduction

Hepatitis B is a serious infectious disease that is the major cause of death from cirrhosis and liver cancer complications. Globally, the disease is thought to have infected 257 million individuals [1]. The majority of cases were found in Asia and Africa, where 68% of the population had positive hepatitis B virus serological results and 8–15% had chronic infections [1–3].

Indonesia is one among the nations in the South East Asian Region (SEAR) with the highest hepatitis B endemicity [1, 3, 4]. Indonesia Basic Health Research (RISKESDAS) in 2013 reported that hepatitis B represents for 7.1% or approximately 18 million of the general population [4]. According to the Indonesian Health Profile 2018, the prevalence of hepatitis B in pregnant women in Indonesia was 1.88% [5]. This is a significant issue because pregnant women who are infected with the hepatitis B virus risk vertical transmission to their unborn child. Infection during pregnancy is linked to a 90% increased risk of chronic hepatitis B, compared to a risk of less than 5% in adults. Around 90% of children infected with the hepatitis B virus during their first year of life develop a chronic infection, with a 25% chance of dying from cirrhosis or liver cancer [6–8]. One of the most crucial parts of breaking the hepatitis B transmission chain is preventing vertical transmission.

The placenta serves as a mechanical and immunological barrier, keeping out pathogens from infecting the fetus [6, 9–14]. The interaction between placental trophoblasts and maternal decidual immune cells is essential for a successful pregnancy because it allows the embryo to develop in the uterus while the mother's immune system develops strategies to tolerate it and coordinates a cell migration surveillance process to recognize and respond to invading microorganisms. Several studies have revealed that placental trophoblast cells act as macrophages that respond to antigens by expressing toll-like receptors, which are homologous proteins that act as Pattern Recognition Receptors (PRRs). Toll-like receptors (TLRs) attach to antigenic structures or Pathogen Associated Molecular Patterns (PAMPs), triggering an immune response [9, 13, 15–20].

Holmlund was the first to detect TLR2 expression in the placenta in a study published in 2007. Expression of TLR2 was found in trophoblast cells, decidual cells, and mesenchymal cells in the mature placenta in this investigation [19, 20]. However, there is currently a scarcity of material outlining the mechanism by which the placenta protects against infections such as hepatitis B virus infection. The expression of TLR2 in the placenta of mothers with hepatitis B virus infection was investigated in this study, as well as its relation to the detection of hepatitis B virus in umbilical cord blood as a marker of intrauterine hepatitis B virus exposure.

## 2. Materials and Methods

### 2.1. Study Population

From December 2019 to October 2020, a cross-sectional observational study was undertaken in Wahidin Sudirohusodo Hospital, Hasanuddin University Hospital, and the teaching hospital network of the Department of Obstetrics & Gynecology, Faculty of Medicine, Hasanuddin University, Indonesia. The Ethical Committee for Health Research of Faculty of Medicine, Wahidin Sudirohusodo Hospital, and Hasanuddin University Hospital authorized this study (Ref. number 1216/UN4.6.4.5.31/PP36/2019).

Pregnant women who gave birth in the study area provided written informed consent before they participated in this study and were counseled and screened for HBsAg. The inclusion criteria comprised a serologically proven HBV infection (HBsAg positivity). Patients with autoimmune disease or coinfected with hepatitis C virus and human immunodeficiency virus were excluded from this study, as well as patients receiving HBV treatment and women who have had invasive procedures during pregnancy, who are at risk of placental leakage, such as amnioinfusion and amniocentesis.

### 2.2. Specimen Collection

After a completely general, systemic, and obstetrical examination, maternal sera that were HBsAg-positive found by rapid test at the initial screening were reconfirmed using HBsAg immunoassay (VIDAS HBsAg BioMérieux SA, Marcy l'Etoile, France). Additional venous blood samples were collected from those with positive screening test results for confirmatory HBsAg testing and subsequent testing for additional HBV markers (HBsAg titer, HBeAg, anti-HBs, and anti-HBc) on the Monolisa TMHBeAg Ag-Ab PLUS (Biorad, France) immunoassay according to the manufacturers' instructions.

The delivery method was chosen based on obstetric considerations. Cord blood samples were obtained shortly after the baby was born for qualitative HBV DNA testing using the automated CobasTaqmanTM HBV Test (Roche Diagnostics, Indianapolis, USA) according to the manufacturer's instructions. The presence of HBV DNA in cord blood was taken as evidence of intrauterine exposure to the hepatitis B virus.

Placental tissue samples were obtained and sent to the laboratory shortly after the placenta was delivered. Immunohistochemistry was used to examine the expression of TLR2 protein in the placenta using CD282 (TLR2) Monoclonal Antibody (TL2.1), Functional Grade, eBioscience™. The number of positive trophoblast cells was evaluated on a scale of + to +++ for TLR staining evaluation. (+, <25% of villous trophoblast cells stained; ++, 25%–75% of villous trophoblast cells stained; +++, >75% of villous trophoblast cells stained). If TLR2 scores 2 and 3 are achieved, the expression is categorized as high, and if scores 0 and 1 are obtained, the expression is classified as low.

### 2.3. Statistical Analysis

Using Statistical Package for Social Sciences (SPSS) ver 21.0 software (SPSS Inc., Chicago, IL, USA), the collected data were analyzed using the chi-square test. A *P* value <0.05 was considered significant.

## 3. Results

59 samples were collected during the study period, with the majority of the study samples from mothers aged 20–35 years (79.7%), multigravida (54.2%), mothers giving birth at term gestational age (74.6%), and mothers with the most vaginal delivery mode (79.7%), as seen in Table 1. The findings of laboratory testing utilizing the enzyme-linked immunosorbent assay (ELISA) method revealed that chronic hepatitis B infection had the highest seroprevalence of hepatitis B (62.7%). There were 19 samples that tested positive for HBsAg but were negative for anti-HBc and anti-HBs antibodies (32.2%). These are unusual serologic indicators that can be used to diagnose extremely early hepatitis B infection, chronic hepatitis B infection without anti-HBc seroconversion, or hepatitis B vaccine outcomes.

We evaluated viral load levels as one of the parameters impacting maternal-to-fetal transmission and discovered that most levels in the sample were <5.3 log 10 IU/ml (88.1%). Viral load was not detected in 15 samples (25.4%). The presence of HBV DNA in the cord blood implies intrauterine exposure to hepatitis B virus infection [21]. Hepatitis B virus was found in 69.5% of umbilical cord blood samples.

**Figure 1:**
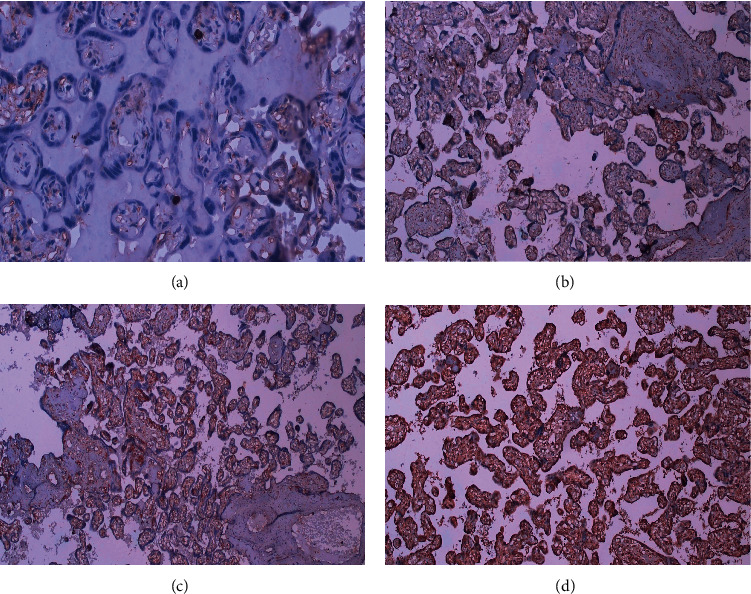
Immunohistochemical staining for TLR2 in placentas of mothers with hepatitis B. (a) TLR2 score of 0 (no villous trophoblast villi stained). (b) TLR2 score of 1 (<25% villous trophoblast villi stained). (c) TLR2 score of 2 (25–75% villous trophoblast villi stained). (d) TLR2 score of 3 (>75% villous trophoblast villi stained).

TLR2 expression was detected in placental tissue using an immunohistochemistry technique. TLR2 expression was predominantly identified in syncytiotrophoblast and cytotrophoblast cells in the placentas of mothers with hepatitis B virus infection (Figure 1). Expression of TLR2 in the placenta with the highest score of 3 was reported in mothers aged 20–35 years (75%), multigravida (58.3%), and mothers with term pregnancies (70.8%).

The results of maternal HBV DNA detection, umbilical cord HBV DNA detection, and TLR2 scoring in placental tissue were compared. Of 59 samples of HBsAg-positive mothers, we discovered 6 undetected HBV DNA. The remaining 53 samples were then statistically analyzed to determine the relation between HBV DNA detection and TLR2 in placental tissue (Table 2).

The chi-square test revealed that there was no relation between placental TLR2 expression and intrauterine hepatitis B virus exposure, with a *p* value of 0.730 and an OR of 0.650 (95% CI: 0.173–2.440).

## 4. Discussion

Indonesia is one of the countries with the highest hepatitis B endemicity [1,2,22]. According to the 2018 Indonesian Health Profile, the prevalence of hepatitis B among pregnant women nationwide was 1.88%, with a prevalence of 2.51% in the province of South Sulawesi where this study was conducted [5]. This study included 59 women who had hepatitis B infection when they delivered. The majority of those who took part were between the ages of 21–35 (79.7%). These data are confirmed by the findings of Dwivedi et al. (2011), who found that the age group of 21–25 years had the highest prevalence of hepatitis B, followed by the age group of 26–30 years [23]. This is in line with the recommended age for giving birth, which is when a woman is in her reproductive years.

Cheung et al. categorized the vertical transmission of hepatitis B into three gestation periods: (1) during conception (germ-line infection); (2) throughout pregnancy (maternal blood contamination or transplacental transmission), and (3) during delivery (rupture of membranes and vaginal delivery). In cases of chorioamnionitis, preterm labor, or the use of birthing aids, the chance of transmission is increased. Positive HBeAg status and high HBV DNA levels are linked to the rate of transmission via these three routes [24, 25].

In this investigation, it was discovered that 69.5% of all pregnant women with hepatitis B infection had intrauterine hepatitis B virus exposure. Chronic hepatitis B was the most common serological profile among the patients (62.7%), with 40.5% testing positive for HBeAg.

Both immune cells, such as hepatocytes, liver nonparenchymal cells, dendritic cells, and nonimmune cells, express toll-like receptors. TLR expression and function are reduced during HBV infection, according to several studies [26]. Preiss et al. were the first to report that HBeAg-positive chronic hepatitis B patients had lower TLR2 expression in hepatocytes, Kupffer cells, and monocytes than HBeAg-negative chronic hepatitis B patients and controls. TNF-induction in hepatic cells was inhibited by HBeAg expression [27]. On PCR testing, Chen et al. found that TLR mRNA and protein levels were significantly lower in chronic hepatitis B patients with Peripheral Blood Mononuclear Cells (PBMC). Expression of TLR2 was identified in the PBMCs of individuals with chronic hepatitis B who had impaired cytokine production. TLR2-mediated Mitogen-Activated Protein Kinase (MAPK) pathway, which indirectly causes monocytes to release IL-10, is not blocked by HBsAg [28, 29]. As a result, TLR2 expression and function appear to be major targets of viral suppression, whereas HBV evolved to evade innate immune system surveillance by targeting the PRR signaling pathway.

Cooper et al. investigated the mechanism of HBcAg-induced proinflammatory cytokine induction and discovered that the nucleocapsid HBcAg rich in arginine is a bonafide macrophage activator and a cytokine-inducing factor mediated by TLR2 [30]. TLR stimulation by Pathogen Associated Molecular Patterns (PAMPs) activates the Myeloid Differentiation Primary Response Gene 88 (MyD88) and TLR-Domain-Containing Adapter-Inducing Interferon-*β* (TRIF) pathways in liver cells, producing proinflammatory cytokines, chemokines, and interferons (IFNs). HBV replication can be inhibited in two ways: (1) by activating antiviral mechanisms via the intracellular Mitogen-Activated Protein Kinase (MAPK) and Nuclear Factor Kappa-Light-Chain-Enhancer of Activated B Cells (NF-kB) pathways and (2) by stimulating the expression of Immune Serum Globulins (ISGs) and activating antiviral activity in hepatocytes. In addition, chemokines and inflammatory cytokines boost T cell proliferation and enhance the antiviral action of CD 8+ T cells, which are HBV-specific effector cells. As a result, TLR suppresses HBV by activating both the innate and adaptive immune systems [18, 31].

The presence of HBV DNA in the umbilical cord of HBV DNA-positive mothers suggests that placental function as an immune barrier has been compromised. Hepatitis B virus transplacental transmission is a complicated and multifaceted process that is regulated by several circumstances. In this study, we found no association between placental TLR2 expression and intrauterine hepatitis B virus exposure. The probability of vertical transmission of hepatitis B infection is affected by both obstetric and virological factors at the same time. A viral load of HBV DNA >5.3 log10 IU/ml has previously been reported as the main risk of perinatal transmission [32]. Even more, infants born to mothers with positive hepatitis B e antigen (HBeAg) or high viral load are more likely to fail active-passive immunoprophylaxis [21]. These infections' competitive strategies for evading host defense and crossing the placental barrier into the fetal compartment are not entirely known and are challenging to set up.

The limitation of the study was the small sample size and the unavailability of long-term follow-up in the infant. The hepatitis B virus itself is transmitted in a complex and multifactored manner. The placenta as an immunological barrier, along with other variables considered to have a role in preventing viruses from reaching the fetus, needs to be studied further.

## 5. Conclusion

TLR2 is predominantly identified in placental syncytiotrophoblast and cytotrophoblast cells; however, TLR2 expression is not associated with intrauterine exposure of hepatitis B virus.

## Figures and Tables

**Table 1:** Sample characteristics.

Characteristics	*n* = 59
Age (*n* (%))	
<20 years old	5 (8.5)
20–35 years old	47 (79.7)
>35 years old	7 (11.9)
Parity (*n* (%))	
Primigravida	27 (45.8)
Multigravida	32 (54.2)
Gestational age (*n* (%))	
Preterm	8 (13.6)
Term	44 (74.6)
Postterm	7 (11.9)
Placental disorders (*n* (%))	
Placenta previa	1 (1.7)
Placental abruption	0
Preeclampsia	2 (3.4)
Premature rupture of the membrane (*n* (%))	3 (5.1)
Fetal distress (*n* (%))	1 (1.7)
Delivery mode (*n* (%))	
Vaginal delivery	47 (79.7)
Cesarean section	12 (20.3)
Maternal serological factors	
HBsAg titer (*n* (%))	
<1000 IU/ml	36 (61)
>1000 IU/ml	23 (39)
HBV DNA (*n* (%))	
Negative	7 (11.8)
Positive	52 (88.1)
HBeAg (*n* (%))	
Negative	43 (72.9)
Positive	16 (27.1)
Anti-HBs (*n* (%))	
Negative	56 (94.9)
Positive	3 (5.1)
Anti-HBc (*n* (%))	
Negative	21 (35.6)
Positive	38 (64.4)
Viral load (*n* (%))	
<5.3 log 10 IU/ml	52 (88.1)
≥ 5.3 log 10 IU/ml	7 (11.8)
Intrauterine exposure of hepatitis B (n (%))	
HBV DNA cord blood negative	18 (30.5)
HBV DNA cord blood positive	41 (69.5)
TLR2 expression in placenta tissue (n (%))	
Score 0	5 (8.5)
Score 1	15 (25.4)
Score 2	15 (25.4)
Score 3	24 (40.7)

**Table 2:** Association of TLR2 expression in the placenta and the detection of HBV DNA.

Placental TLR2 expression	HBV DNA detection (*n* (%))		Odds ratio	95% CI	*P*
	HBV DNA maternal (+), HBV DNA cord blood (+)	HBV DNA maternal (+), HBV DNA cord blood (-)			
High score	7 (58.3)	28 (68.3)	0.650	0.173–2.440	0.730
Low score	5 (41.7)	13 (31.7)			

High score = scores 2-3; low score = scores 0-1.

## Data Availability

Derived data supporting the findings of this study are available from the corresponding author upon request.
